# Human Papillomavirus: An Old New History

**DOI:** 10.3390/pathogens14101043

**Published:** 2025-10-14

**Authors:** Nicole West, Valentina Boz, Nunzia Zanotta, Carolina Cason, Giuseppina Campisciano, Alessandra Casuccio, Daniele Gianfrilli, Teresa Maria Assunta Fasciana, Giuseppina Capra, Maria Cristina Salfa, Franz Sesti, Barbara Suligoi, Francesca Valent, Laura Brunelli, Manola Comar

**Affiliations:** 1 Department of Medical, Surgical, and Health Sciences, University of Trieste, 34127 Trieste, Italy; nicolewest.tlb@gmail.com (N.W.); giuseppina.campisciano@burlo.trieste.it (G.C.); 2 Unit of Clinical, Treatment and Immunodeficiency Research, Department of Pediatrics, Institute for Maternal and Child Health, IRCCS “Burlo Garofolo”, 34137 Trieste, Italy; valentina.boz@burlo.trieste.it; 3 Department of Advanced Translational Microbiology, Institute for Maternal and Child Health-IRCCS Burlo Garofolo, 34137 Trieste, Italy; nunzia.zanotta@burlo.trieste.it; 4 Section of Hygiene, PROMISE Department, University of Palermo, 90127 Palermo, Italy; alessandra.casuccio@unipa.it; 5 Department of Experimental Medicine, Sapienza University of Rome, 00185 Rome, Italy; daniele.gianfrilli@uniroma1.it (D.G.); franz.sesti@uniroma1.it (F.S.); 6 Department of Health Promotion, Mother and Child Care, Internal Medicine and Medical Specialities, University of Palermo, 90127 Palermo, Italy; teresa.fasciana@unipa.it (T.M.A.F.); giuseppina.capra@unipa.it (G.C.); 7 AIDS Operational Centre, Department of Infectious Diseases, Istituto Superiore di Sanità, 00161 Rome, Italy; mariacristina.salfa@iss.it (M.C.S.); barbara.suligoj@iss.it (B.S.); 8 SOC Accreditation, Quality, and Clinical Risk, Friuli Centrale Healthcare University Trust, 33100 Udine, Italy; francesca.valent@asufc.sanita.fvg.it (F.V.); laura.brunelli@asufc.sanita.fvg.it (L.B.); 9 Department of Medicine, University of Udine, 33100 Udine, Italy

**Keywords:** human papillomavirus, adolescents, vaccination, screening, microbiome, prevention

## Abstract

Human papillomavirus (HPV) represents the most common sexually transmitted infection worldwide and a major public health challenge. Nearly all sexually active individuals will acquire HPV during their lifetime, with the highest prevalence observed in adolescents and young adults shortly after sexual debut. More than 200 genotypes have been described, ranging from low-risk types, mainly responsible for benign lesions, to high-risk types, which are associated with cervical, anogenital, and head and neck cancers. While most infections are transient and spontaneously cleared by the immune system, persistent high-risk HPV can lead to precancerous lesions and malignant transformation, often in synergy with other sexually transmitted pathogens or in the context of microbiome imbalance. The introduction of vaccines and advanced screening technologies has substantially modified prevention strategies. Vaccination coverage remains heterogeneous, with persistent gaps particularly among males due to cultural, social, and educational barriers. Schools are increasingly recognized as strategic environments to promote awareness, sex education, and gender-neutral vaccination. Innovative approaches such as microbiome modulation, therapeutic vaccines, and liquid biopsy biomarkers are emerging as promising perspectives. This review aims to provide an updated overview of HPV epidemiology, clinical impact, prevention strategies, and future frontiers, with special attention to adolescents as a priority target group.

## 1. Introduction

The links between human papillomavirus (HPVs) and cervical cancer were first demonstrated almost 50 years ago [1]. Currently, advanced biotechnological approaches have improved the knowledge of host–virus interplay, highlighting the key role of host immune response, microbiome homeostasis, and co-infection with sexually transmitted pathogens. Moreover, technologies such as liquid biopsies, DNA methylation triage, and detection of HPV integration have implemented traditional screening methods [2,3,4].

HPV is a DNA tumor virus belonging to the Papillomaviridae family and today, more than 200 genotypes have already been characterized and sequenced [5,6,7]. Each genotype of HPV acts as an independent infection, with differing carcinogenic risks. HPV subtypes can be categorized into Low Risk (LR) variants, which encompass types 6, 11, 4, 40, 42, 43, 44, 54, 61, 70, 72, and 81, associated with benign lesions including genital, anal, oral, or throat warts, and into High Risk (HR) variants consisting of types 16, 18, 31, 33, 35, 39, 45, 51, 52, 56, 58, 59, 68, 73, and 82, where HPV-16 and -18 cause the majority of HPV -related cancers including cervical, anogenital, and head–neck cancers [5,6,8]. The clinical signs were strongly related to host immune response. Basically, while LR-HPV infections resolve spontaneously in a couple of years, HR-HPV establishes a persistent infection that overcomes the immune system’s defenses, forming precancerous lesions that can progress to cancer over time [5,6,8,9].

HPV infection is considered one of the most common sexually transmitted infections (STIs) worldwide with significant implications for public health [10]. Epidemiological data report that 80% of sexually active individuals will be infected by HPV during their lifetime. Age-specific analysis revealed a high prevalence of HPV peaks in adolescence and among young population under 25 years old after first sexual activity [6,11]. Young men over the age of 15 years reported a high prevalence of infection [6,11], representing an important and often silent reservoir for viral transmission. HPV prevention acts to target the population through proactive intervention strategies including screening, vaccination, information campaigns, education and awareness about HPV and STIs coinfections implicated in HPV transmission and clinical progression [12].

The HPV vaccine is offered to adolescents between 9 and 14 years of age, or in three doses to individuals aged 15 years or older [13] and, in some countries, this schedule has been adopted for both males and females since 2018 [14]. Although many countries have introduced a national program [15], the overall coverage is still not optimal. Vaccine safety concerns and a lack of HPV knowledge still represent the main barriers, especially for males. To overcome this cultural gap, efforts about educational programs are recently proposed to schools that represent the best place to introduce a pedagogical approach for sex education, prevention, and social responsibility [11]. The aim of this review is to explore the actual global magnitude of HPV impact on public health systems, with particular attention to adolescents and focus on the emerging potential alternatives for HPV prevention and diagnosis.

## 2. Materials and Methods

This narrative review was conducted to summarize current evidence on human papillomavirus (HPV) epidemiology, clinical manifestations, prevention strategies, and future perspectives, with a particular focus on adolescents. A non-systematic literature search was performed in the main biomedical databases (PubMed, Scopus, Web of Science) for publications in English up to August 2025. Search terms included combinations of “human papillomavirus”, “HPV epidemiology”, “HPV vaccine,” “screening,” “microbiome,” “sexually transmitted infections,” and “therapeutic vaccine.” Additional references were identified from the bibliographies of relevant articles and official reports from international health agencies such as the World Health Organization (WHO) and the European Centre for Disease Prevention and Control (ECDC).

Given the narrative nature of this work, no predefined protocol or systematic review methodology (e.g., PRISMA) was applied. Selection of articles was based on relevance, originality, and contribution to the understanding of HPV infection and prevention. Both original research and review articles were considered, along with recent epidemiological reports and policy documents. Particular attention was given to studies addressing HPV burden in adolescents, vaccine acceptance, and innovative diagnostic or preventive strategies. The evidence was synthesized to provide an integrated and updated overview, aiming to highlight knowledge gaps and future research directions.

## 3. Epidemiology

According to the latest World Health Organization (WHO) data, HPV infection contributes to 5% of all cancers worldwide each year, with approximately 626,000 women and 69,400 men diagnosed with HPV-related cancers. Infection with HR-HPV, particularly HPV types 16 and 18, accounts for about 70% of cervical cancers globally. Cervical cancer is the fourth most common cancer among women worldwide, with an estimated 660,000 new cases and 350,000 deaths in 2022.

Among women, the cervix was the most commonly affected site (46%), followed by the anus (20%), vulva (17%), oropharynx (14%), and vagina (3%). In men, the majority of HPV-associated cancers occurred in the oropharynx (82%) [16]. Mortality data from Europe shows nearly 26,000 deaths in 2020, making cervical cancer the tenth leading cause of death in women and the third most common cause of death in women aged 15–44 [17]. Global pooled prevalence for genital HPV infection among men was 31% for any HPV and 21% for HR-HPV where HPV-16 is the most common HPV oncogenic and preventable type. HPV prevalence in men peaked in the group aged 25–29 years and remained high until at least age 50 years. Prevalence in the group aged 15–19 years was also high, suggesting that young men are being infected rapidly following first sexual activity. The age profile of infection in women is different from that of men, showing a prevalence peaks soon after first sexual activity that declines with age, and a slight rebound after age 50–55 years [18]. Among extragenital HPV-related cancer, most of them occurred in the oropharynx (82%) [11].

In response to these data, the WHO launched a global strategy in 2020 to address cervical cancer as a public health issue. The strategy aims for 90% of girls to be fully vaccinated with the HPV vaccine by age 15, 70% of women to undergo screening by the ages of 35 and 45, and 90% of women diagnosed with cervical disease to receive appropriate treatment (90% of women with precancerous conditions treated and 90% of women with invasive cancer treated). This is a global strategy to accelerate the elimination of cervical cancer as a public health problem [19]. The WHO has clearly recognized the efficacy of the HPV vaccine on a global scale. However, the actual population impact and vaccination coverage are highly heterogeneous, with the most consistent data on cancer reduction coming from high-income countries in Europe, North America, and Oceania, where vaccination programs are best established. Studies conducted in Australia, Northern Europe, and the United States of America, ten years after the introduction of the first HPV vaccines around 2007, found a significant decrease in HPV infections of genotypes 6, 11, 16, and 18 in both vaccinated young women and the general population, and a consequent decrease in the incidence of genital warts [20,21]. Updated data is also sourced from countries such as Sweden. A Swedish population-based cohort study assessed the effectiveness of the quadrivalent HPV vaccine and found a significant reduction in high-grade cervical lesions. The efficacy of the vaccine was found to be greater in girls who received the vaccine at an earlier age (before the age of 15) than in those who received three doses of the vaccine at an older age [22]. Evidence from large population-based studies, such as the Swedish one [22], supports ongoing policy decisions in several countries; for example, the United Kingdom recommends a single dose for girls and boys up to age 25 [23] and Spain has recommended a single dose up to age 18, starting in 2024 [24]. The objective of these changes is to maximize vaccination coverage by simplifying the program and reducing costs. In Northern Italy, a pilot study, published in 2021 by Fappani et al., also observed a positive trend in the depletion of HPV genotypes included in vaccines, both in young women eligible for the national vaccination program and in the total number of circulating HPV genotypes, suggesting a broad impact of the vaccination for the targeted types involved [25]. Nevertheless, disparities in access to healthcare persist, and this has a significant impact on the incidence of cervical cancer, especially among women from racial and ethnic minorities and in rural areas, as well as among women of low socioeconomic status [26].

## 4. Clinical Manifestations and Co-Infection with STI

HPV pathogenesis begins in the host cell, where HPV releases its viral genome which is endocytosed in the nucleus of the same cell to replicate its DNA in synchrony and to express viral genes [5,6,27]. Translated genes are mainly those encoding the oncoprotein E6 and E7, which are linked to viral pathogenic activity and are crucial for the transformation of infected cells as they are involved in the regulation of the host cell’s cycle [5,6,28,29,30]. The clinical manifestations range from minor issues such as warts to serious conditions like tumors. Anogenital warts are typically asymptomatic, although their size and anatomical location can cause discomfort or pruritus [5].

The vast majority of anogenital warts, approximately 90%, are caused by non-oncogenic HPV types 6 or 11. They are typically flat, papular, or pedunculated growths that commonly manifest in specific anatomical locations, including the vaginal introitus, the foreskin of the penis, the cervix, the vagina, the urethra, the perineum, the perianal skin, the anus, and the scrotum. In rare cases, oncogenic HPV types 16, 18, 31, 33, and 35 can also be identified in these lesions [31]. A rare but highly disabling disease is the recurrent respiratory papillomatosis affecting the upper aerodigestive tract of both children and young adults.

Papillomavirus typically presents as exophytic nodules, predominantly in the larynx, although they may also occur in the nasopharynx, tracheobronchial tree, and lung parenchyma. In some cases, lesions may resolve spontaneously. However, in rare instances, there is a potential for the lesion to evolve into squamous cells carcinoma. In clinical practice, recurrent respiratory papillomatosis typically presents with nonspecific symptoms indicative of airway involvement, including chronic cough, hoarseness, wheezing, voice changes, stridor, and chronic dyspnea [32,33,34]. In some instances, these lesions resolve spontaneously, while in other cases, surgical intervention is recommended to prevent disease progression [35].

In a small proportion of women, the virus can establish a persistent infection, probably due to the synergistic effect of a suboptimal host-dependent immune response which may initiate the process of carcinogenesis. Genotypes 16 and 18 are primarily associated with the development of carcinomas in the cervix, the anogenital region (vulva, vagina, penis, and anus), and the head and neck (mouth, tonsils, pharynx, and larynx) [36,37]. The most common type of cancer due to a persistent HR-HPV infection is cervical cancer and, along with other risk factors, it can lead to low-grade squamous intraepithelial lesions (L-SIL), which include mild dysplasia known as CIN 1 (cervical intraepithelial neoplasia grade 1). This can then progress to high-grade squamous intraepithelial lesions (H-SIL). H-SIL is a progressive lesion that can evolve into moderate dysplasia (CIN 2) and then severe dysplasia (CIN 3) [38,39,40,41]. The most clinically encountered cervical tumors are squamous cell carcinoma and adenocarcinoma.

Recent studies have shown that the presence of cervical lesions related to HPV infection increases the risk of acquiring other STIs. It was reported that in women, persistent HPV infection raises the likelihood of contracting HIV, [42] and at the same time, an HIV-positive woman has a higher risk of acquiring HPV infection, especially due to high-risk types [5,43]. The co-infection with persistent high-risk HPV and *Chlamydia trachomatis* (CT) has been suggested as a contributing factor to the advancement of cervical cancer in women. The ability of CT in activating chronic inflammation and producing damage to epithelial integrity played a key role to support persistence of HPV infection and cells’ transformation [42,44]. Moreover, recent studies revealed the role of *Escherichia coli* in infecting HPV-16 cervical lesions that seem to cooperate in the severity of disease [45]. Another pathogenic mechanism has recently been considered: the presence of herpes simplex virus 2 (HSV2) RNA in cervical biopsies that are abnormal on cytological examination [46] and the higher risk of HPV co-infection in HSV-positive women compared to HSV-negative women [46,47,48]). The HPV strains most closely associated appear to be HPV-18 and HPV-58, rather than HPV-16 [47]. As early as 1976, G.R.B. Skinner hypothesized the involvement of HSV2 in the initial stages of cellular transformation of uterine cancer, but not in its progression, coining the term “hit and run” mechanism [49]. HSV2 is thought to attack the integrity of the cervical epithelium, facilitating the entry of the HPV virus and giving rise to an inflammatory process that aids the carcinogenesis process [46,47,48]. The optimal spread of HPV-16, on the other hand, has been observed to be facilitated by microlesions in the epithelium of the cervix caused by infection with *Trichomonas vaginalis*, a flagellated protozoan that induces an environment rich in proinflammatory cytokines that promote the spread of the HPV virus [50,51].

## 5. Extragenital HPV-Associated Cancers

Although genital cancers are in both genders, the incidence of the most common and frequent disease associated with HPV infection, head and neck squamous cell carcinoma (HNSCC), has increased more rapidly in recent years, particularly in Europe and the United States [52,53]. HNSCC refers to a class of squamous cell carcinoma that arises in the mucosal surfaces of the head and neck region, which can develop in various sites of the upper aerodigestive tract, including the oral cavity: oropharynx, larynx, and hypopharynx [5,54].

Although alcohol and tobacco abuse is found as the main risk factor in the onset of tumors in the upper sites of the aerodigestive tract [55], a high proportion of these cancers, especially in the oropharyngeal portion, can be attributed to HPV infection [5,54,55], mainly to HPV-16, with a higher prevalence in men than in women [5,54].

However, an important aspect can be observed when comparing patients with HPV-related HNSCC and patients with the same disorder not related to infection. Patients with non-HPV-related HNSCC show a less favorable prognosis and a lower response to treatment compared to those positive for HPV [5,54].

Although it is clear that HPV positivity plays a key role in the development of HNSCC, the biological behavior of these tumors differs depending on the site of infection.

Furthermore, it has been observed that even low-risk HPV genotypes may be associated with malignant lesions in certain non-cervical anatomical sites. As reported by Lima da Silva et al., HPV6 and HPV11 may be involved together or individually in the development of laryngeal and penile cancer. However, they are not sufficient to trigger the disease completely, but the presence of co-carcinogens or other risk factors is required for epithelial carcinogenesis to occur. These data imply that the use of vaccines that act against a broader spectrum of HPV genotypes, such as the nonavalent vaccine, is also useful also for the prevention of extragenital cancer [56].

## 6. Testing and Screening

Specific diagnosis and screening programs are two essential tools in the management of HPV infections. The primary diagnostic tools have been cytology (Pap test) and histology, along with nucleic acid amplification tests (NAATs), which are currently the most common method for detecting HPV and are considered the gold standard [57]. NAATs can detect the presence of HPV DNA or messenger RNA (mRNA geno hpv) in a variety of samples, including cervical swabs, anal and buccal swabs, or saliva. PCR-based tests are widely used due to their high sensitivity, specificity, and ability to detect multiple HPV genotypes simultaneously [3,54,58].

The most common routine for cervical cancer screening is based on the Pap test and the HPV-DNA test. A Pap test looks for precancerous cell changes on the cervix that might develop into cervical cancer if not treated appropriately, while an HPV test looks for the HPV virus, which can cause cell changes on the cervix [58]. Current guidelines recommend that women between the ages of 25 and 30 should be tested with cervical cytology alone (Pap test), and screening should be performed every 3 years. The HPV-DNA test is offered to women between the ages of 30 and 64 every 5 years if the result is negative. There is no approved screening test for HPV in men, but HPV testing may be required for men who are at high risk of infection, such as those who are immunocompromised or those who have sex with other men [59]. Despite WHO recommendations in terms of prevention, only one-third of women aged 30–49 have been screened at least once in their lifetime. This percentage varies across global regions, with the percentage of access to treatment and prevention options below 10% in low-income and lower-middle-income countries which also accounted for the highest burden of disease [60].

The most recent studies in terms of screening are focused on developing alternative techniques to PCR-based tests, but with the same sensitivity and specificity given the low sensitivity and specificity of cytological tests, which are not very reproducible, require highly skilled specialists, and are not the optimal choice for detecting small precancerous lesions [61]. This is to achieve a reduction in terms of costs, equipment, and highly specialized personnel to increase screening coverage even in the poorest areas of the world [58]. One of these approaches is called isothermal amplification techniques (IATs), which operate at constant temperature and are often shorter than PCR. The most engaged IAT is a loop-mediated isothermal amplification (LAMP), which provides for the use of Bst polymerase and a group of four to six primers to rapidly amplify either DNA or RNA, a common approach to study bacterial and viral nucleic acid [54,58]. Therefore, LAMP could be a good choice for fast determination of HPV infection, but it is highly sensitive to contamination and primers need to be created with a special software and to be tested before using them [54,58]. According to the need for a quick, simple, and cost-effective technique, we can transfer attention on the research of specific HPV-DNA with reverse dot/line blot or on the protein detection through lateral flow assay (LFA). The first method provides the hybridization of gene-specific oligonucleotide probes, coated on a predefined dot or line, with complementary target DNA, allowing the contemporary identification of more HPV types together. Instead, the second one, LFA, is based on a paper analytic platform to detect the presence or the absence of target analytes, which are released based on the principle of an ELISA. If the analyte is present, it binds to a labeled antibody and this complex moves along the strip to the detection zone where it is recognized by immobilized antibodies or antigens in the test or control line; if there is no analyte, the line does not appear.

This technique could be coupled with PCR or IAT amplification, and this combined approach has been used to detect genomic DNA of different HPV genotypes [58]. The last approach is focused on the innovative CRISPR-Cas-based system and the implementation of Cas12a, which targets a specific gene of HPV, helping the recognition of HPV-16 and 18 in a variety of clinical samples, such as plasma and anal swabs. CRISPR-Cas system has a great sensitivity and specificity with a relatively inexpensive manner, but it requires a lot of precision in the design of guide RNAs [54,58]. To address the high cost of reagents and the risk of contamination associated with nucleic-acid-based methods, and to find an approach for larger-scale screening programs, a commercial pre-denaturing solution, PharmaDirect, is proposed, which allows for direct PCR without the need for DNA extraction in cervical swab samples [62]. PharmaDirect shows a high level of specificity and sensitivity, the latter especially for HPV-16 detection, but less so in cases of infection with multiple viral genotypes concurrently [62]. The final goal of research into new HPV screening techniques will be the development of point-of-care tests to enable increasingly simple and rapid diagnosis, even in areas of the world with fewer economic and healthcare resources [58,63]. In this context, nanotechnology offers great potential for the development of point-of-care diagnostic tools. The aim of this technology is to identify the latest generation of electrochemical and photoelectrochemical biosensors for faster, more sensitive, and more specific HPV testing [64]. In addition, in their 2025 review, He et al. focused on the effectiveness of semiconductor nanocrystals, called Quantum Dots, used as fluorescent markers linked to biosensors to increase the specificity and sensitivity of HPV DNA identification [64].

Not only are the techniques in the field considered for the improvement of screening programs, but also the procedure of test sample collection and its type.

To perform screening tests, the collection of samples can be made as self-sampling [54,63]. Self-sampling is a safe and simple approach which can increase screening rates due to better privacy and personal comfort compared to traditional sampling [54,63]. Moreover, self-sampling enables greater screening coverage, especially in low-resource areas where infrastructure and healthcare workforce are very limited. Indeed, thanks to this evidence, in 2021, the WHO included self-sampling in its guidelines for cervical cancer screening, and it has already been introduced as a sample collection protocol in 35% of the 139 countries that have an official screening recommendation plan [60,65,66]. In addition, DNA methylation technology is a molecular triage test providing accurate results across settings without comprehensive cervical cancer screening programs, exploiting methylation changes in several host gene promoters that we know to be associated with HPV infection and its progression [67]. The most studied genes are *CADM1*, *FAM19A4*, *MAL*, and *miR124-2*, and *FAM19A4* shows a similar methylation profile, even if the sample is self-collected, compared to that which is picked in specialized centers [67]. Another methylation panel has been included in clinical practice by the WHO guidelines, GynTect, which targets six host genes: *ASTN1*, *DLX1*, *ITGA3*, *RXFP3*, *SOX17*, and *ZNF671*. This panel is highly specific in identifying CIN3 lesions [68].

The methylation test could be used for primary non-invasive screening, referring HPV-positive/methylation-positive women to immediate treatment and negative women to follow-up after an appropriate number of years using a single sample [67,69].

Recent research focused on seeking new biomarkers for early diagnosis or prognostic outcome in patients with HPV-mediated cancer. In this context, a recent study showed that the presence of circulating tumor DNA (ctDNA) in the blood could be a possible robust biomarker using digital polymerase chain reaction or NGS technology. Chennareddy et al. (2025) highlight the role of ctDNA to monitor the treatment response. It was reported that both tumor resection and chemotherapy were responsible for a decrease in ctDNA levels, and its complete clearance at the end of the chemotherapy cycle is associated with a higher chance of recovery [4,70,71,72].

## 7. Prevention

Currently, there is no established therapeutic intervention for individuals infected with HPV, but various therapeutic protocols are available for clinical management [66,73]. The prophylactic use of vaccines against HPV to prevent the development of cancer has proven to be an effective method. HPV vaccines are based on the L1 capsid protein, which assembles into virus-like particles (VLPs). These VLPs induce antibodies that neutralize specific HPV types and are antigenically identical to HPV virions, enabling effective immune responses [74]. The comprehension of HPV-induced carcinogenesis, together with the accessibility of VLPs, has facilitated the creation of numerous prophylactic vaccines against HPV, which are summarized in Table 1. Gardasil^®^ (Merck, Sharp & Dome) (Merck & Co., Whitehouse Station, NJ, USA) is a quadrivalent vaccine targeting HPV-6, HPV-11, HPV-16, and HPV-18. It provides protection against genital warts caused by HPV-6 and HPV-11 and high-risk HPV types linked to cervical cancer. Cervarix^®^ (GlaxoSmithKline, Rixensart, Belgium) is a bivalent vaccine targeting HPV-16 and HPV-1. Clinical trials have demonstrated its efficacy against high-grade cervical intraepithelial neoplasia (CIN3) and a strong safety profile [75,76,77,78,79]. Gardasil 9^®^ (Merck, Sharp & Dome) (Merck & Co., Whitehouse Station, NJ, USA) is a nonavalent vaccine licensed in 2014 that targets HPV-6, HPV-11, HPV-16, HPV-18, HPV-31, HPV-33, HPV-45, HPV-52, and HPV-58 [80,81]. Cecolin^®^ (Xiamen Innovax Biotechnology, Xiamen, China), a bivalent vaccine using *Escherichia coli* to produce HPV-16 and HPV-18 L1 VLPs [82]. Cecolin^®^ was pre-qualified by the WHO in 2021. Recently a recombinant bivalent HPV vaccine (Shanghai Zerun Biotechnology, a subsidiary of Walvax Biotechnology, Shanghai, China) targeting HPV-16 and HPV-18 has been pre-qualified by the WHO for use in 2022 [83].

Following the European Centre for Disease Prevention and Control (ECDC) directives, many European countries had introduced HPV vaccination for females and some of them had organized catch-up programs. Recently, the HPV program has been extended to males and implemented in gender-neutral vaccination [85,86]. Each vaccine provides specific protection against targeted subtypes and demonstrates some degree of cross-protection against non-vaccine HPV subtypes [87]. Vaccination induces significantly higher antibody titers against specific HPV types than those observed in naturally infected individuals. Notably, individuals vaccinated before the age of 16 exhibit higher antibody titers than older adolescents and adults [88] and this reflects the importance of vaccine administration before the onset of sexual activity to maximize the effect of vaccine protection, which is easier to achieve when people have not yet been exposed to HPV infections [5]. Recent data indicate an increase in single-dose HPV vaccine coverage among girls aged 9–14 years, rising from 20% in 2022 to 27% in 2023 [88]. Due to the possibility of accurate screening programs, the cervical cancer elimination strategy established by the WHO provides coverage targets for scale-up by 2030 to 90% of all adolescent girls, twice-lifetime cervical screening to 70%, and treatment of pre-invasive lesions and invasive cancer to 90% [89].

## 8. Educational Programs

To improve vaccination coverage and move closer to the goals given by WHO for 2030, it is crucial to implement education programs starting from adolescents in terms of sex education, knowledge about HPV and STIs, and prevention strategies including the importance of vaccination [10,90,91]. In terms of education, the school remains the ideal place to implement training programs, thus enabling better interaction with health services [92]. On this issue, in 2024, Brunelli et al. recently published an innovative multidisciplinary model taking place in upper and secondary schools.

The proposed training program was supported by a team of experts who train identified volunteers in schools to conduct permanent peer meetings about STIs and HPV to raise awareness of sexual and reproductive health issues, including vaccination, to implement the effectiveness of the training among students, while adults (parents and teachers) will participate in distance and face-to-face trainings [90]. Two interesting aspects emerged recently concerning adolescent self-awareness and gender-neutral vaccination [92]. In many states, parental consent is needed to vaccinate adolescents, who cannot intervene in choices for their own health. For this reason, it is referred to as self-consent, which must be preceded, however, by appropriate education to enable adolescents to make concealed health choices [10,15,93]. In addition, the possibility of having training programs in schools highlights the disparity between males and females in the opportunity to receive the HPV vaccine, introducing the concept of gender-neutral vaccination. Only a minority of countries with an HPV vaccine program included males in vaccine administration [15]. In 2017, Italy was the first European country to introduce universal vaccination for both males and females [90].

Vaccination campaigns were designed only for girls because of the major side effects that HPV infection can give them, first and foremost cervical cancer, and that they would also reflexively protect boys through herd immunity if vaccination coverage in females is high enough [15,94,95]. Unfortunately, the effect of herd immunity is currently not achievable given the low overall vaccination rate, and it was suggested that it is more cost-effective to increase the level of coverage in girls than to offer the vaccine to boys as well [15]. To note, men are also susceptible to HPV oncogenic infections that usually can progress to anal and penile cancers, demonstrating the undeniable importance of adherence of the vaccine program as prevention strategy for boys as well.

## 9. Future Directions

### 9.1. Microbiome

**Figure 1:**
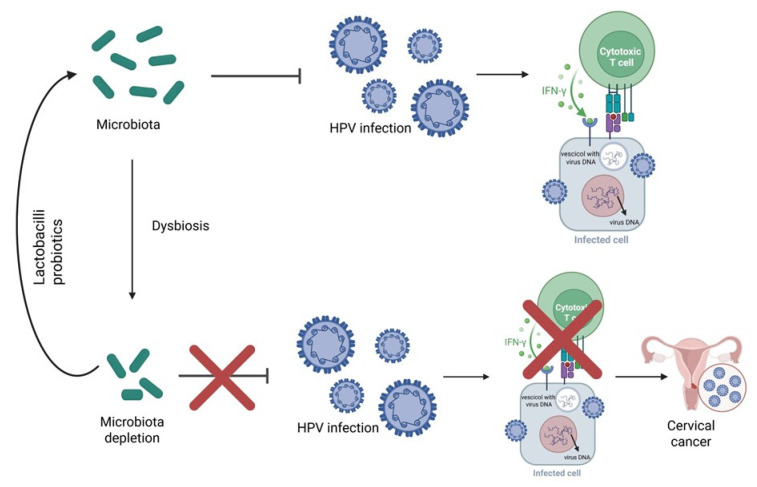
Interplay between human microbiota and HPV. Vaginal microbiota inhibits the interaction with possible pathogens, such as HPV, promoting the activation of cytotoxic T cells which produced molecules, like IFN-γ, that target and eliminate HPV-infected cells. However, in the case of dysbiosis, there is no inhibition and no consequent promotion of the activation of the immune response. This way, HPV infection can develop and lead to cervical cancer [98]. The introduction of Lactobacilli probiotics restores the homeostasis of the vaginal microbiota, allowing it to inhibit HPV infection (Figure created in https://BioRender.com).

The next-generation sequencing (NGS) technology and bioinformatics have allowed us to unveil the interplay between human microbiota and HPV in terms of infection, establishment, persistence, and disease progression. Basically, it has been demonstrated that bacterial species composition can modulate the susceptibility of infections, patients’ outcomes, and more interestingly, the host immune response to infection [96]. The vaginal dysbiosis represents a dysregulated and functionally impaired microbiome that tends to promote chronic low-grade inflammation, immune dysregulation, oxidative stress, DNA damage, and metabolic rewiring. In addition to dysbiosis, the host response, including cellular and cytokine markers of inflammation, increased susceptibility for STIs, HR-HPV and progression to cervical carcinogenesis [97] (Figure 1).

It is proven that vaginal microbiota can be modulated to restore local homeostasis and to promote beneficial effects to host through the HPV clearance and cytological abnormalities [99,100]. The predominance of Lactobacilli species is a sign of homeostasis in the vaginal microbiome, but if the balance shifts in favor of anaerobic bacteria, this leads to a state of dysbiosis, mainly caused by a high rate of colonization by *Gardnerella vaginalis* [51,101,102]. This pathogenic bacterium adheres to a cell’s surface, altering the mucous barrier and inducing the progression of HPV infection and the consequent formation of CIN [51]. The increase in anaerobic bacteria in the vaginal microbiome also facilitates interaction with other infectious agents such as *Trichomonas vaginalis*. Together, these co-infections increase the likelihood of infection by the HPV virus, its persistence, and its progression to cervical cancer [51,101]. In this context, Lactobacilli are the most common probiotics for microbiota modulation, acting to prevent pathogens infections through bacterial competition and the release of inhibitory factors [103,104]. At the same time, enhanced immune response and reduction in inflammation were observed [105], suggesting its possible future application in terms of personalized medicine. Along with probiotics, a-glucans are also effective in helping the immune system clearance HPV infection [102]. An approach that is still experimental and under development involves transplanting microbiota from healthy donors. This approach includes a careful preliminary selection phase to avoid transplanting microbiota containing drug-resistant microorganisms, hidden pathogens, or traces of sperm [102].

### 9.2. Therapeutic Vaccine

To have an effective weapon against HR-HPV infections that is non-invasive and requires less specialized technical support, a new generation of vaccines based on transgenic technology to engineer live bacteria and probiotics, enabling them to produce and deliver both preventive and therapeutic antigens, is being proposed. These vaccines are called mucosal vaccines [106,107]. In this context, a promising vaccine vector is Gram-positive *Lactococcus lactis* due to its safety profile, lack of endotoxic lipopolysaccharides (LPS), and cost-effectiveness [108]. Another possibility in using these bacterial vectors is the ability to induce the expression of certain proteins that function as antigen delivery carriers, activating the host’s immune system. This type of vaccine can directly target the reproductive tract mucosa to deliver antigens and elicit a protective local immune response [109]. For example, the engineered drug BLS-M07—which expresses the HPV-16 E7 antigen on the surface of *Lactobacillus casei*—is administered orally. Park et al. conducted Phase I and IIa clinical trials to assess its efficacy and safety in patients with CIN3 (Cervical Intraepithelial Neoplasia Grade 3). In this approach, the HPV E7 protein on the *Lactobacillus* surface was recognized by antigen-presenting cells (APCs), which in turn activated cytotoxic T cells [110]. The trial results demonstrated a significant increase in serum HPV-16 E7-specific antibodies (75%), with no patients experiencing treatment-related adverse events of grade 3 or higher throughout the study [110].

## 10. Discussion and Conclusions

HPV knowledge and vaccine acceptance vary across different countries. Safety concerns are still the main barrier to vaccination, and lack of HPV, vaccine knowledge, and social responsibility has been identified, especially in males [111]. Moreover, the idea that HPV vaccination may encourage sexual activity is widespread among the general population. HPV vaccination coverage rates and parental acceptance are still a subject of debate, although several initiatives have been recently implemented by experts with particular attention to adolescent and young population, based on multidisciplinary tools including educational, parental, scientific, and political resources. Nevertheless, HPV vaccine acceptance varies by target population’ characteristics where socioeconomic background represents a key bias. The need for “tailored” interventions, designed to respond to personal, epidemiological, and local public health concerns would be offered to the target population to reduce social inequalities and thus decrease the impact on health governance. In addition, strategies of interventions oriented towards adolescents seem to be more effective. In this sense public health interventions with the additional components of education, social tools, and remained-based strategies appeared to be positively accepted.

## Figures and Tables

**Table 1:** HPV vaccines: overview of current commercially available vaccines against HPV [84].

Commercial Name	Type of Vaccines (Target Genotypes)	Intended Recipients	Age of Administration	Vaccine Boosters	Vaccine Boosters Beyond Age of Administration
Cervarix	Bivalent vaccines (HPV-16, HPV-18)	Female and male	9–14	2-dose (5–13 months apart)	3-dose (1–2.5 and 5–12 months)
Cecolin	Bivalent vaccines (HPV-16, HPV-18)	Female	9–14	2-dose (6 months apart)	3-dose (1–2 and 5–8 months)
Walrinvax	Bivalent vaccines (HPV-16, HPV-18)	Female	9–14	2-dose (6 months apart)	3-dose (2–3 and 6–7 months)
Gardasil	Quadrivalent vaccines (HPV-6, HPV-11, HPV-16, HPV-18)	Female and male	9–13	2-dose (6 months apart)	3-dose (1–2 and 4–6 months)
Cervavax	Quadrivalent vaccines (HPV-6, HPV-11, HPV-16, HPV-18)	Female and male	9–14	2-dose (6 months apart)	3-dose (2 and 6 months)
Gardasil9	Nonavalent vaccines (HPV-6, HPV-11, HPV-16, HPV-18, HPV-31, HPV-33, HPV-45, HPV-52, HPV-58)	Female and male	9–14	2-dose (5–13 months apart)	3-dose (1–2 and 4–6 months)

## Data Availability

Not applicable.
